# Risk of cardiovascular mortality, stroke and coronary heart mortality associated with aircraft noise around Congonhas airport, São Paulo, Brazil: a small-area study

**DOI:** 10.1186/s12940-021-00746-7

**Published:** 2021-05-13

**Authors:** Aina Roca-Barceló, Adelaide Nardocci, Breno Souza de Aguiar, Adeylson G. Ribeiro, Marcelo Antunes Failla, Anna L. Hansell, Maria Regina Cardoso, Frédéric B. Piel

**Affiliations:** 1 UK Small Area Health Statistics Unit (SAHSU), Department of Epidemiology & Biostatistics, School of Public Health, Imperial College London, London, UK; 2 Department of Environmental Health, School of Public Health, University of São Paulo, São Paulo, Brazil; 3 Epidemiology and Information Department, Municipal Health Secretariat of São Paulo, São Paulo, Brazil; 4 Centre for Environmental Health and Sustainability, University of Leicester, Leicester, UK; 5 Department of Epidemiology, School of Public Health, University of São Paulo, São Paulo, Brazil; 6 National Institute for Health Research Health Protection Research Unit (NIHR HPRU) in Environmental Exposures and Health, London, UK

**Keywords:** Aircraft noise, Cardiovascular disease, Stroke, Mortality

## Abstract

**Background:**

Noise pollution is increasingly recognised as a public health hazard, yet limited evidence is available from low- and middle-income countries (LMIC), particularly for specific sources. Here, we investigated the association between day-night average (L_dn_) aircraft noise and the risk of death due to cardiovascular disease (CVD), stroke and coronary heart disease (CHD) at small-area level around São Paulo‘s Congonhas airport, Brazil during the period 2011–2016.

**Methods:**

We selected 3259 census tracts across 16 districts partially or entirely exposed to ≥50 dB aircraft noise levels around the Congonhas airport, using pre-modelled 5 dB L_dn_ noise  bands (≤50 dB to > 65 dB). We estimated the average noise exposure per census tract using area-weighting. Age, sex and calendar year-specific death counts for CVD, stroke and CHD were calculated by census tract, according to the residential address at time of death. We fitted Poisson regression models to quantify the risk associated with aircraft noise exposure, adjusting for age, sex, calendar year and area-level covariates including socioeconomic development, ethnicity, smoking and road traffic related noise and air pollution.

**Results:**

After accounting for all covariates, areas exposed to the highest levels of noise (> 65 dB) showed a relative risk (RR) for CVD and CHD of 1.06 (95% CI: 0.94; 1.20) and 1.11 (95%CI: 0.96; 1.27), respectively, compared to those exposed to reference noise levels (≤50 dB). The RR for stroke ranged between 1.05 (95%CI: 0.95;1.16) and 0.91 (95%CI: 0.78;1.11) for all the noise levels assessed. We found a statistically significant positive trend for CVD and CHD mortality risk with increasing levels of noise (*p* = 0.043 and *p* = 0.005, respectively). No significant linear trend was found for stroke. Risk estimates were generally higher after excluding road traffic density, suggesting that road traffic air and noise pollution are potentially important confounders.

**Conclusions:**

This study provides some evidence that aircraft noise is associated with increased risk of CVD and CHD mortality in a middle-income setting. More research is needed to validate these results in other LMIC settings and to further explore the influence of residual confounding and ecological bias. Remarkably, 60% of the study population living near the Congonhas airport (~ 1.5 million) were exposed to aircraft noise levels > 50 dB, well above those recommended by the WHO (45 dB), highlighting the need for public health interventions.

**Supplementary Information:**

The online version contains supplementary material available at 10.1186/s12940-021-00746-7.

## Background

The health impact of noise pollution is a growing public health concern. Beyond the direct risk of noise to the auditory system, there is growing evidence of a wide range of physical and cognitive complications associated with the role of noise as a non-specific stressor [1–3]. An in-depth study conducted by the European Environmental Agency suggested that one in five Europeans were exposed to harmful noise levels and estimated that environmental noise was responsible for one million disability-adjusted life years (DALYs) in Europe only [4].

Noise levels are usually considered excessive when they exceed 65 dB (dB) during the day and 55 dB at night. A recent meta-analysis published to support the 2018 WHO Environmental Noise Guidelines for the European region [5] found sufficient and strong evidence linking road traffic noise to cardiovascular outcomes, particularly ischemic disease. Nonetheless, the quality of evidence for other noise sources, including aircraft noise, was found to be moderate to very low, accentuating the need for more and better quality evidence [6]. Accordingly, the WHO’s guidelines were conservative, recommending levels below 45 dB and 40 dB for average daily noise (L_dn_) and night-time exposure (L_night_), respectively for aircraft noise. To date, no such guidelines exist for non-European countries.

A recent systematic review summarizing current evidence on environmental noise and cardiovascular and metabolic effects [6] estimated aggregated relative risks of 1.05 (95% CI 0.95–1.17), 1.09 (95% CI: 1.04–1.15) and 1.14 (95% CI 1.03–1.25) per 10 dB L_DEN_ for hypertension, ischemic heart disease and stroke in relation to aircraft noise. Again, most of the evidence identified came from studies conducted in European countries. For example, the HYpertension and Exposure to Noise near Airports (HYENA) study found a positive association between excess risk of hypertension and long term exposure to aircraft noise around six major European airports [7]. Evrard and colleagues found similar results for night noise around French airports where exposure-response relationship for hypertension was studied [8, 9]. In the UK, the Small Area Health Statistics Unit (SAHSU) conducted a small-area study of the risk of cardiovascular disease (CVD), stroke and coronary heart disease (CHD) associated with aircraft noise around Heathrow airport, the second busiest airport in the world by international passenger traffic [10], finding an association with CVD, CHD and stroke mortality and hospital admissions. Beyond Europe, Peters and colleagues reviewed evidence on aviation noise and cardiovascular health in the United States [11].

The issue of noise pollution in low- and middle-income countries (LMICs) has only recently started to gain more attention [12]. Assessing the health impact of aircraft noise in densely populated megacities in these countries would help quantifying the risks and the benefits of public health interventions to reduce exposure. It is of particular concern as large numbers of people with potential increased vulnerability to noise, including children, the elderly and individuals with chronic disease live in megacities [13, 14]. Additionally, those from deprived areas and from ethnic minority backgrounds, have been found to have higher exposures in European inner cities [14], which can contribute to further increases in health inequalities.

The city of São Paulo in Brazil provides a good example of the noise pollution challenges faced by megacities in LMICs. Congonhas airport - the second busiest airport in Brazil (22.7 million passengers in 2019) [15] - is located right in the middle of a densely populated area of São Paulo. Although studies have estimated exposure to aircraft noise pollution in São Paulo [16, 17], health impacts have so far not been assessed. According to the Brazilian Civil Aviation Regulation n° 161, areas with aircraft noise above 65 dB require restrictions in the land use and activities allowed, as well as the implementation of strategies to reduce ground noise levels [18].

This study aims to investigate the association between aircraft noise levels and CVD, stroke and CHD mortality risk among people living in the vicinities of the Congonhas airport, in São Paulo city, Brazil between 2011 and 2016.

## Study methods

All procedures described here were approved by the ethics committee of the University of São Paulo (CAAE: 08337619.6.0000.5421) and conducted in accordance with Brazilian Law 13,709/2019 on protection of personal data.

### Study area and population

**Fig. 1:**
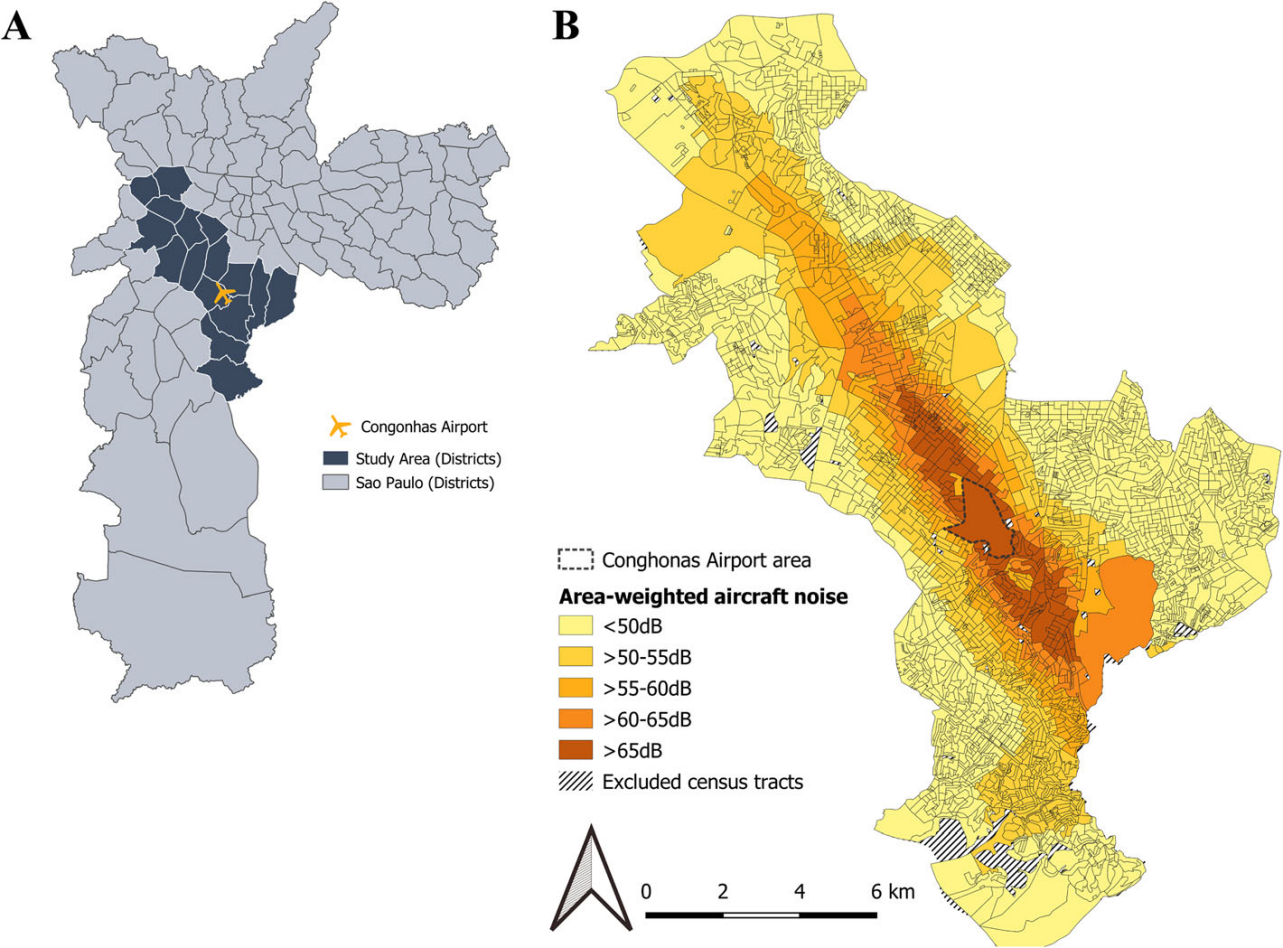
Study area (districts) and exposure bands based on area-weighted aircraft noise per census tract. **a**. Study area (dark grey) around Congonhas Airport (orange aircraft symbol) selected for being partially or entirely exposed to aircraft noise ≥50 dB. The light grey area represents the extent of the Municipality of São Paulo, Brazil. The boundaries shown are those of the districts; **b**. Area-weighted aircraft noise at census tract level across the study area. Hatched areas correspond to census tract with a population smaller than five individuals (e.g. parks), which have been excluded from the study

We defined the study area based on existing noise contours for the Congonhas airport (see 2.3 below). We included 16 districts (171.8 km^2^) partially or entirely exposed to aircraft noise (Fig. 1a). Neighbourhoods were then defined using census tracts, the smallest geographic unit for which mortality and covariate data were available. Our study area covered 3259 census tracts (average: 542 inhabitants), excluding those with < 5 inhabitants or not populated, such as parks (Fig. 1b).

Population estimates at census tract level were not available for inter-censal years. Therefore, annual census-tract level population by age and sex were modelled from the official annual age- and sex-specific projected population data for administrative districts provided by the State-wise System for Data Analysis (SEADE) Foundation [19]. These estimates are sensitive enough to capture intra-urban variability [20], and therefore were considered adequate for our study area. For this purpose, we took the annual age- and sex-specific population change rate of each district and applied it to the 2010 census tracts’ population as provided by the Brazilian Institute of Geography and Statistics (IBGE) [21]. This approach relies on the assumption that population changes at the census tract level followed similar trends to those at the district level. Given that our study area covered a densely populated area, substantial urban development and population growth were unlikely to have occurred. In the absence of more granular data, this was the best available approximation.

### Outcome data

Data on deaths among population aged over 20 years old occurring between 2011 and 2016 in the study area were obtained from the Epidemiology and Information Department of the Municipal Health Secretariat of São Paulo (CEInfo/SMS-SP) [22]. Data were provided stratified by age, sex, year and underlying cause of death. For this study, we only considered the following underlying causes of death, as defined by the International Classification of Disease 10th edition (ICD-10) [23]: (i) CVD (ICD10: Chapter I); (ii) stroke (ICD10: I61, I63-I64), and (iii) CHD (ICD-10: I20-I25) [23]. Death certificates in Brazil are compulsory, and forms are filled in by physicians, with death causes coded according to the ICD-10. Health departments of each municipality collect information from the death certificates and record it periodically in the online national database, DATASUS [24]. Over recent years, the quality of death records in Brazil and São Paulo has improved remarkably providing the necessary information for defining specific underlying cause-of-death [25, 26].

### Exposure data

Noise levels in Brazil are monitored and regulated by the Brazilian airport Infrastructure Company (INFRAERO, 2018). They are modelled using the integrated noise model (INM) version 7.0d developed by the US Federal Aviation Administration (FAA) and approved by the Brazilian National Civil Aviation Agency (ANAC) [27]. The model combines information on all aircrafts operating in 2017 including their flight patterns and times of arrival and departure as well as height, speed, and engine power. The modelled ground noise levels represented the day-night average sound level (L_dn_); the average equivalent sound level during 24 h, with a penalty of + 10 dB for night-time noise (10 pm to 7 am) [28]. Flight restrictions are in place between 11 pm and 6 am in Congonhas airport, therefore reducing the number of night flights. Nonetheless, overnight flights still represented 8% of the total operating flights (~ 496 operations/day) and so, were included in the modelling.

INFRAERO data were available as L_dn_ contours categorized as follows: < 65, 65–70, 71–75, 76–80, 81–85, and > 85 dB **(**Fig. S1A, Additional File 1). As most of these bands fell within the perimeter of the airport, contours ≥65 dB were merged. To gain more granularity in lower noise contours, we used additional noise contours developed by Slama and colleagues using the same INM approach for 50, 51–55, 56–60, 61–65 and > 65 dB (Fig. S1B, Additional File 1). The contours, obtained directly from the authors (Slama, pers. com.), were based on flight information and supported by measurements taken at several monitoring stations around the airport. The INM software used to model noise has been accredited for use in national aircraft noise monitoring and used repeatedly in health impact assessments of exposed population [29] including in the landmark HYENA study [7]. The model validity was therefore considered strong enough to use its outputs in this epidemiological study.

Each of the contours represented a simplification of a continuous distribution of aircraft noise values across the contour area, where the upper and lower bands of the contour represented the minimum and maximum aircraft noise estimates in the distribution. We took the midpoint between the minimum and maximum values as a summary of the noise distribution for each band. As noise data are on a log scale (dB), we calculated the antilog values first.

We used an area weighting approach (Eq. 1) to harmonize the noise level at each census tract included in the study area for belonging to a district intersect any of our noise contours. Where *AWnoise*_*i*_ is the area-weighted noise value for census tract *i*; *area*_*ij*_ is the area of the census tract *i* intersecting the noise contour *j*; *area*_*i*_ is the total area of census tract *i*, and *antilog(noise)*_*j*_ is the antilog value of the noise for the contour *j*.

1 $$ {AWnoise}_i=\sum \limits_i^j\left(\left(\frac{area_{ij}}{area_i}\right)\times anti\log {(noise)}_j\right) $$

Firstly, we intersected the census tracts with the aircraft noise contours layer and estimated the area intersecting each contour. For a census tract, the proportion of area intersecting a given noise contour was used to weigh the contribution of that contour in the overall noise level for that census tract. Finally, weighted noise values were aggregated for each census tract and re-logged. The final output was an area-weighted annual average aircraft noise (dB) for each census tract. We classified these census tracts in the following 5 dB groups for congruency with the original contours categorization, i.e. ≤50 dB, > 50–55 dB, > 55–60 dB, > 60–65 dB, and > 65 dB.

### Covariates

We accounted for the following covariates in our modelling approach: area-level socioeconomic development, ethnic composition, area-level smoking, and noise and air pollution from road traffic. The spatial distributions of these covariates are shown in Fig. S2A-F, Additional File 1.

#### Socioeconomic development

To reflect the socioeconomic development of the census tracts in our study area, we used an adaptation of the Human Development Index (HDI), developed by the United Nations Development Program (UNDP) [30]. This synthetic measure of the degree of economic development and life quality across countries has been recurrently used to represent deprivation at finer spatial scales in Brazil, including municipalities (e.g. the Municipal Human Development Index, MHDI) [31] and census tracts in São Paulo [32]. It considers three dimensions: life expectancy, income and education, grouped by a geometric mean. As life expectancy is a health indicator, we have re-calculated the MHDI scores excluding that dimension, as a sensitivity analysis, using the same methodology. In addition to the continuous scores, we classified the census tracts in quintiles based on the distribution of the standard and modified MHDI scores across the study area. We evaluated the correlation between the standard and modified indicators using Pearson’s correlation coefficient of their scores (0.9973, *p*-value< 0.005, Fig. S3, Additional File 1) and Spearman’s rank order correlation for the quintile classification (0.9827, p-value< 0.005). The spatial distribution of both the continuous score and the quantile classification were also assessed (Fig. S4, Additional File 1), and showed similar patterns. Hence, we used the standard MHDI quantile classification to adjust the main model. The modified MHDI quantile classification was used in sensitivity analysis.  

#### Ethnic composition

Census tract level ethnic composition was obtained from the 2010 census [21]. We calculated the proportion of Black (census term: *Preto*) and Mixed ethnicity (*Pardo* or *Brown*) groups combined, and the proportion of East Asians (*Amarelo* or *Yellow*) across our study area and in each census tract*.* No Indigenous (*Indigeno*) population was recorded in our study area. We then classified each census tract in one of three categories based on their deviance from the study area average using two different cut off points: the average (Black and Mixed population: 19.34%; Asian population: 4.09%)  and two-fold the average proportion of each group. This resulted in three categories: (i) lower than the average, as an indicator of low prevalence of a certain ethnic group; (ii) between the average and twice the average, and (iii) higher than twice the study area’s average, indicating a high concentration per area of people from the ethnic group under study.

#### Smoking prevalence

Data on individual smoking or prevalence of smoking across the study area were not available. However, over 90% of the lung cancer cases can be explained by cumulative smoking exposure [33]. Hence, lung cancer mortality (ICD-10: C33-C34) can be used as an indirect measure or proxy for cumulative smoking exposure. This approach has been used previously to estimate the global burden of mortality associated to tobacco [33, 34] and as an adjustment variable in epidemiological studies [10, 35]. Hence, we used smoothed lung cancer mortality (ICD-10: C33-C34) relative risk (RR) estimates between 2010 and 2016 modelled at the census tract level, as a proxy measure for area level smoking. We modelled this risk as a Poisson distribution using the Besag–York–Molliè (BYM) [36] model presented in Eq. 2.1 and 2.2. This modelling approach takes into account both structured and unstructured spatial dependencies. Where *O*_*i*_ is the number of observed and *E*_*i*_ the expected number of deaths at census tract *i*; *λ*_*i*_ is the risk of lung cancer death defined by *n*_*i*_ and calculated as intercept (*β*_0_) added to the sum of the spatially structured residual (*u*_*i*_), modelled using the intrinsic conditional autoregressive (iCAR) specification coupled with an exchangeable random effect, and the unstructured residual (*v*_*i*_) modelled using exchangeability among all the census tracts.

S2.1 $$ {\displaystyle \begin{array}{c}{O}_i\sim Poisson\left({\lambda}_i{E}_i\right)\\ {}{n}_i=\log \left({\lambda}_i\right)={\beta}_0+{u}_i+{v}_i\end{array}} $$

Where *u*_*i*_ and *v*_*i*_ are modelled as follows:

S2.2 $$ {\displaystyle \begin{array}{c}{u}_i\mid {u}_{-i}\sim Normal\left({\mu}_i+\frac{1}{\aleph_i}\sum \limits_{j=1}^n{a}_{ij}\left({u}_j-{\mu}_j\right),{s}_i^2\right)\\ {}{v}_i\sim Normal\left(0,{\sigma}_v^2\right)\end{array}} $$

The main assumption underlying this model is that the spatial distribution of smoking prevalence is highly associated to the spatial distribution of lung cancer mortality in our area of study. An exception to this assumption is the presence of one or more additional risk factors, which either (i) have a dissimilar spatial distribution to smoking, and/or (ii) are risk factors also for the outcome of interest in our main model (i.e. CVD, coronary disease and/or stroke). Both situations were deemed unlikely in our study area (see Discussion section for more detail).

#### Traffic noise and air pollution

To account for road traffic noise and air pollution levels, we used road traffic density as a proxy. Census tract road traffic density was defined as the sum of all vehicle counts per hour (vehicle/hour) along the length (meters) of all road segments (m^2^) falling within a given census tract. The dataset included routine data collected by the Traffic Engineering Company of São Paulo (CET) with additional traffic counts performed by the co-authors for a previous study using the same methodology [32] The streets were classified according to their function in traffic distribution as: expressway, arterial-1, arterial-2, arterial-3, collector-1, collector-2 and local. All traffic information was entered into a geo-coded street database. Traffic density was calculated for each census tract using the software ArcGIS ArcInfo 9.3 [37]. A full description of the methodology can be found elsewhere [32]. Data were categorized in quintiles based on the traffic density distribution across the study area, with the 5th quintile representing the highest road traffic density (Table 1).

**Table 1:** Census tract, population and cause-specific death counts (and %) per exposure and covariate levels

					Health outcome					
		Census tracts	Population 2010^a^		CVD		Stroke		CHD	
		n	n	%	n	%	n	%	n	%
**Exposure**	**Noise levels**									
	≤ 50 dB	1835	2,049,934	58.04	12,377	56.42	2055	57.07	4721	55.82
	> 50–55 dB	749	790,422	22.38	4751	21.66	822	22.83	1820	21.52
	> 55–60 dB	316	326,106	9.23	2096	9.56	325	9.03	823	9.73
	> 60–65 dB	205	207,354	5.87	1431	6.52	216	6.00	569	6.73
	> 65 dB	154	158,394	4.48	1281	5.84	183	5.08	524	6.20
**Covariates**	**MHDI 2010**									
	Q1 - the poorest	652	855,980	24.23	3726	16.99	656	18.22	1349	15.95
	Q2	652	809,818	22.93	5324	24.27	903	25.08	2040	24.12
	Q3	652	659,400	18.67	5023	22.90	787	21.86	1919	22.69
	Q4	652	614,414	17.39	4248	19.37	670	18.61	1691	20.00
	Q5 - the wealthiest	651	592,598	16.78	3615	16.48	585	16.25	1458	17.24
	**Total traffic density** (vehicle/hour/m^2^)									
	Q1 - lowest	652	722,466	20.45	2454	11.19	433	12.02	905	10.70
	Q2	652	796,238	22.54	4694	21.40	767	21.30	1846	21.83
	Q3	652	744,686	21.08	5096	23.23	833	23.13	1944	22.99
	Q4	652	652,568	18.47	4762	21.71	754	20.94	1849	21.86
	Q5 - highest	651	616,252	17.45	4930	22.47	814	22.60	1913	22.62
	**Black and Brown population**									
	< average	1871	1,799,222	50.94	12,364	56.36	1963	54.51	4937	58.38
	Average - 2-fold average	515	608,980	17.24	4513	20.57	750	20.83	1676	19.82
	> 2-fold average	873	1,124,008	31.82	5059	23.06	888	24.66	1844	21.80
	**Smoking proxy**									
	≤ 0.85	123	429,194	12.15	898	4.09	109	3.03	357	4.22
	> 0.85, 0.95	455	1,636,602	46.33	2668	12.16	458	12.72	1016	12.01
	> 0.95, 1.05	1503	1,119,786	31.70	10,030	45.72	1655	45.96	3907	46.20
	> 1.05, 1.15	985	215,012	6.09	6933	31.61	1171	32.52	2649	31.32
	> 1.15	193	131,616	3.73	1407	6.41	208	5.78	528	6.24
	**Total**	**3259**	**3,532,210**		**21,936**		**3601**		**8457**	

^a^ 2010 population estimates provided as this was the year of the census

Note: *CVD* Cardiovascular disease. *CHD* Coronary heart disease. *MHDI Índice de Desenvolvimento Humano Municipal* (Municipal Human Development Index). Data sources for mortality data: SIM/SMS-SP; noise levels: INFRAERO and Prof Jules G. Slama (personal communication); Index of Human Development and total traffic density: Ribeiro et al 2019; Black and Brown population: IBGE

### Statistical analyses

Correlations between covariates and noise exposure were assessed using Cramer’s V-square test and bar plots (Figs. S5-S9, Additional File 1). In order to evaluate to risk of death from CVD, stroke and CHD associated with aircraft noise exposure, we fitted a Poisson regression model with a random effect term to account for over-dispersion and residual heterogeneity. For ease of interpretation, we used L_dn_ levels ≤50 dB as the reference category. The model was build using a forward stepwise regression approach, using the Akaike information criterion (AIC) for variable selection. The equation of the fully adjusted model is depicted in Eq.3.

3 $$ {\displaystyle \begin{array}{c}{O}_i\sim P\left({\lambda}_i{E}_i\right)\\ {}\log \left({\lambda}_i\right)={\beta}_0+{\beta}_1{noise}_i+{\beta}_2{Traffic}_i+{\beta}_3{Depriv}_i+{\beta}_4{Ethnos}_i+{\beta}_5{Smoking}_i+{e}_i\\ {}{e}_i\sim N\left(o,{\sigma}_e^2\right)\end{array}} $$

Where:

*O*_*i*_ number of deaths at census tract_i_.

*E*_*i*_, expected number of deaths at census tract_i_, considering the control census tract year, age and sex-specific area population.

*λ*_*i*_ risk, mean number of deaths during the study period in census tract_i_.

*β*_0_ the intercept.

*β*_1_the regression coefficient (fixed effect) of the noise level effect (*noise*_*i*_) for census tract_i._

*β*_2_the regression coefficient (fixed effect) of the total traffic air pollution quintile (*Traffic*_*i*_) for census tract_i._

*β*_3_ the regression coefficient (fixed effect) of the Index of Human Development quintiles (*Depriv*_*i*_) for census tract_i._

*β*_4_ the regression coefficient (fixed effect) of the black and brown proportion variable (*Ethnos*_*i*_) for census tract_i._

*β*_5_ the regression coefficient (fixed effect) of the quintiles of smothered lung cancer risk of death as a smoking proxy (*Smoking*_*i*_) for census tract_i._

*e*_*ij*_ heterogeneity (level-1).

**Fig. 2:**
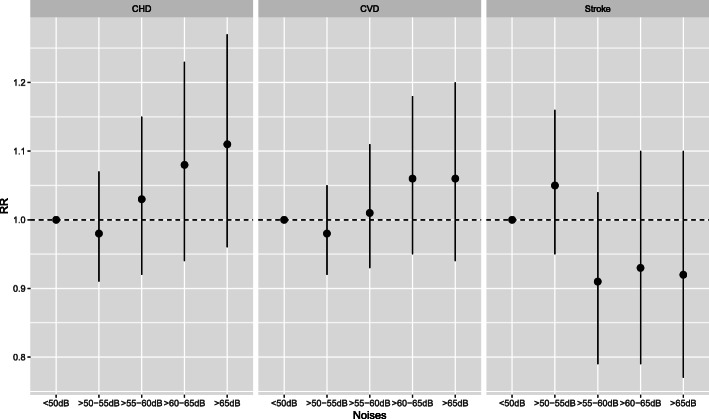
Association between CHD, CVD, and stroke mortality and annual-population weighted average L_dn_ aircraft noise (relative to ≤50 dB) between 2011 and 2016. Adjusted model only shown. Black circles, relative risk estimates; solid likes, the 95%CI

We compared our basic model (BM), adjusted only for age and sex (direct standardization), with our fully adjusted model (AM) which accounted for Black and Mixed ethnicity, standard MHDI 2010, our smoking proxy and road traffic density (Table 2**,** Fig. 2). In addition, we conducted six sensitivity analyses (SA) to test the following: the exclusion of road traffic density (SA1); the use of a modified version of the MHDI to exclude life expectancy (SA2); the incremental inclusion of covariates (SA3–4), and the inclusion of East Asian ethnicity in addition to Black and Mixed (SA5). AIC values for all models are shown in Table S1. We tested for linear trends across noise categories for the two main models (BM and AM) using linear regression. In addition, we estimated the risks per 10 dB increase to facilitate comparison with other studies using the WHO standard methodology (Table S2, Additional File 3).

**Table 2:** Relative risk (RR) and 95% confidence intervals (CI) for the association between CVD, stroke and CHD mortality and annual area-weighted average L_dn_ aircraft noise, between 2011 and 2016 around the Congonhas Airport in São Paulo, Brazil

	CVD				Stroke				CHD			
Noise levels	RR	95% CI		p-trend	RR	95% CI		p-trend	RR	95% CI		p-trend
Basic Model *												
≤ 50 dB	1			0.939	1			0.096	1			0.304
> 50–55 dB	0.96	0.90	1.03		0.99	0.9	1.1		0.95	0.87	1.03	
> 55–60 dB	1.06	0.96	1.17		0.94	0.81	1.08		1.05	0.94	1.18	
> 60–65 dB	0.97	0.87	1.10		0.84	0.7	0.99		0.99	0.87	1.14	
> 65 dB	1.00	0.87	1.14		0.86	0.71	1.03		1.06	0.91	1.23	
Adjusted Model **												
≤ 50 dB	1			**0.043**	1			0.253	1			**0.005**
> 50–55 dB	0.98	0.92	1.05		1.05	0.95	1.16		0.98	0.91	1.07	
> 55–60 dB	1.01	0.93	1.11		0.91	0.79	1.04		1.03	0.92	1.15	
> 60–65 dB	1.06	0.95	1.18		0.93	0.79	1.1		1.08	0.94	1.23	
> 65 dB	1.06	0.94	1.2		0.92	0.77	1.1		1.11	0.96	1.27	
Sensitivity Analysis 1 (excl. Road traffic) ***												
≤ 50 dB	1			0.087	1			0.21	1			**0.02**
> 50–55 dB	1.00	0.94	1.07		1.07	0.97	1.18		1.00	0.92	1.09	
> 55–60 dB	1.05	0.96	1.15		0.94	0.82	1.07		1.07	0.96	1.19	
> 60–65 dB	1.09	0.97	1.22		0.96	0.81	1.13		1.1	0.97	1.26	
> 65 dB	1.08	0.96	1.22		0.93	0.78	1.11		1.13	0.98	1.31	
Sensitivity Analysis 2 (incl. Modified MHDI 2010) ****												
≤ 50 dB	1			**0.029**	1			0.263	1			**0.002**
> 50–55 dB	0.98	0.91	1.04		1.04	0.95	1.15		0.97	0.9	1.06	
> 55–60 dB	1.01	0.92	1.1		0.9	0.79	1.03		1.02	0.91	1.14	
> 60–65 dB	1.05	0.94	1.17		0.92	0.78	1.09		1.06	0.93	1.21	
> 65 dB	1.05	0.93	1.19		0.91	0.76	1.09		1.10	0.95	1.27	

*Basic Model: adjusted for age and sex (direct standardization); ** Adjusted Model: Basic model + smoking proxy + standard MHDI 2010 + Black and Mixed ethnicity + Road traffic density; *** **Sensitivity Analysis 1:** Adjusted model excluding road traffic density, and ****Sensitivity Analysis 2: Adjusted model with the modified MHDI 2010 (i.e. excl. Life expectancy). Other models are described in Table S1, Additional File 2. **Bolded**, the statistically significant p-trend values (i.e. < 0.05)

Note: *CVD* Cardiovascular Diseases; *CHD* Coronary Heart Diseases; *RR* relative Risk; *CI* Confidence Intervals

Analyses were conducted separately for each cause of death considered. All analyses were conducted in STATA Software Version 13.1. and R software Version 3.6.3 .

## Results

Our study area was composed of 16 districts and 3259 census tracts, covering an area of 172km^2^. The average area of the census tracts within our study area was 50.8m^2^, with a standard deviation of 145.0m^2^ and an interquartile range of 38.42m^2^. We found that a total average population of 1,482,276 inhabitants (60% study population) were exposed to aircraft noise > 50 dB. Overall, 21,936 CVD related deaths were recorded in our study area between 2011 and 2016 (i.e. 3656 per year on average). These included 3601 deaths related to stroke and 8457 deaths related to CHD (Table 1). Approximately, 4.5% of the population were estimated to be exposed to the highest level (> 65 dB) of aircraft noise exposure.

According to the standard MHDI, 47.2% of our study area population lived in areas considered amongst the two lowest socioeconomic levels (24.2 and 22.9%, respectively). Deaths were more common in areas belonging to the second and third quintiles for all three causes. Mapping of the standard MHDI scores revealed an overall North-to-South gradient of development across the study area, with the North-Eastern areas being the most developed (Fig. S2A, Additional File 1). The standard and modified MHDI scores were highly correlated (Fig. S3, Additional File 1) and showed a similar spatial distribution (Fig. S4, Additional File 1). A total of 1388 census tracts, representing 49.1% of the population, had a proportion of Black and Mixed population over the average for the study area. Amongst these, 62.9% exceeded 2-fold the average. These areas were located mainly in the southernmost part of our study area (Fig. S2B, Additional File 1). The average road traffic density was 139.8 (interquartile range (IQR): 50.0; 145.8) vehicles/hour/m^2^ within our study area. The total traffic density was the highest in the centre and northern part of our study area (Fig. S2D, Additional File 1). Finally, most of the census tracts showed a smoothed lung cancer mortality risk between 0.95 and 1.05. Finally, Figs. S2E and S2F (Additional File 1) depict the residual relative risk of lung cancer mortality and its uncertainty. The highest risks (> 1.15) seemed to cluster, with low uncertainty, in the centre and south of our study area; whereas low risk areas (< 0.85) were concentrated in the northern part of our study area, albeit with high uncertainty.

Correlations between covariates are shown in Figs. S5-S9, Additional File 1. All the correlations with aircraft noise levels were weak, with V-Cramer estimates always under ≤0.12.Between covariate variables, we observed a relatively high correlation (V-Cramer = 0.70) between categories of Black and Mixed ethnicity population and MHDI 2010 quintiles, with census tracts with a high Black and Mixed ethnicity population percentage often coinciding with the poorest areas (i.e. 1th quintile MHDI 2010). The percentage of East Asians was negatively correlated with the proportion of Black and Mixed ethnicity (V-Cramer = 0.59) and positively correlated with the quintiles of MHDI 2010 (V-Cramer = 0.54) so that, areas with a high proportion of East Asians had lower Black and Mixed ethnicity population percentage, and higher quintiles of MHDI 2010. Correlations among other covariates were weaker (V-Cramer < 0.50).

The AIC values and description of the different models fitted in our forward stepwise model approach can be found in Table S1, Additional File 2**.** The best fit was for the fully adjusted model - AM (AIC_CVD_ = 17,838, AIC_stroke_ = 8778, and AIC_CHD_ = 12,627). Excluding road traffic density from the model (SA1) led to a worsening of the model’s fit. Adjusting for the modified MHDI 2010 index (excl. Life expectancy) (SA2) also worsened the model fit (AIC_CVD_ = 17,853, AIC_stroke_ = 8783, and AIC_CHD_ = 12,639). Models only adjusting for age, sex and MHDI (SA3) or for age, sex, MHDI and smoking (SA4) also performed less well. The addition of the East Asian population covariate (SA5) did not explain much of the residual variability (AIC_CVD_ = 17,840, AIC_stroke_ = 8778, and AIC_CHD_ = 12,632).

The RRs associated with L_dn_ noise exposure for the four models presented are shown in Table 2. The results of the age- and sex-adjusted model only (BM) for CVD and CHD indicated risk estimates oscillating above and below 1 and wide confidence intervals. No significant linear trend was observed (p-trend_CVD_ = 0.939, and p-trend_CHD_ = 0.300). For stroke, risk estimates showed a reduction with increasing noise exposure, only significant for the second to highest level (RR_> 60-65dB_ = 0.84, 95%CI: 0.70;0.99), and a non-significant test for trend (p-trend_stroke_ = 0.096).

After adjusting for all the covariates considered (AM), the risk estimates for CVD and CHD were mostly above 1 and the confidence intervals narrowed slightly (Table 2**,** Fig. 2). Despite none of the risk estimates being statistically significant, we found clear and statistically significant positive trends with increasing noise exposure for CVD and CHD (p-trend_CVD_ = 0.043, and p-trend_CHD_ = 0.005). Risk estimates for stroke also increased after adjustment, yet they remained below one and showed wider confidence intervals. After removing road traffic density, the model (SA1) showed marginally higher risk estimates with slightly broader confidence intervals. The test for trend remained statistically significant for CHD (p-trend_CHD_ = 0.020) but not for CVD (p-trend_CVD_ = 0.087), suggesting that failing to adjust for road traffic related air and noise pollution (here captured through road traffic density) can lead to residual confounding or effect modification by background noise or synergistic/antagonistic effects**,** Fig. 2. This is further supported by the smaller AIC value obtained for SA1. Finally, adjusting for the modified MHDI (excl. Life expectancy, SA2) slightly reduced the effect size, yet it did not modify the overall direction and significance of the effect. Finally, we calculated the risk of death for each outcome per 10 dB increase to facilitate comparisons with other studies (Table S2, Additional File 3). Given that the noise exposure was modelled to capture 5 dB intervals we present the categorical models as our main analysis.

## Discussion

In this cross-sectional small-area study, covering a population of 3,5 million people living near the international Congonhas Airport in São Paulo, Brazil, we show that 60% of the study population (~ 1.5 million) were exposed to aircraft noise levels > 50 dB, well above those recommended by the WHO (45 dB), and provide suggestive evidence of a small dose-response association for CVD and CHD after adjustment for major confounders, with a statistically significant trend for both diseases, but not for stroke. This adds to the growing body evidence of the impact of noise pollution on the health of urban citizens and, to our knowledge, represents the first study to evaluate the effects of aircraft noise on the health of the population living around a major airport in Brazil, or even in a LMIC.

High levels of background noise pollution have previously been reported for São Paulo [38], including in areas surrounding the Congonhas Airport [16]. Noise has been recognised as a risk to human health, primarily based on available occupational and community level evidence [39–41]. The RRs identified in the present study for noise exposures > 65 dB for CVD (1.06, 95%CI: 0.94–1.20) and CHD (1.11, 95%CI: 0.96–1.27) were not statistically significant but consistent with RRs of 1.04 (95%CI: 0.98–1.11) per 10 dB for CHD published in a meta-analysis conducted by the WHO-European region [6], and later updated to 1.03 (95%CI: 0.98–1.09) by Vienneau et al. [42]. If we compare our estimates with specific studies, there exists a large variability in the risk estimates [10, 11, 43–45]. For example, our risks were lower than those found in Hansell et al’s [10] earlier study which reported a RR of death of 1.16 (95%CI: 1.04; 1.29) for CVD, and 1.15 (1.02 to 1.30) for CHD in population living near Heathrow Airport in London, UK, although confidence intervals overlap. Other studies have found no evidence for CVD or CHD but a slight increased risk per 10 dB for stroke and heart failure (HR_stroke_:1.056 and HR_Heart failure_:1.027) [43] while, we did not find any association or statistically significant trend between aircraft noise and stroke in the present study.

The differences observed between our study and others in the literature can be attributed to multiple factors. It is possible that the level of risk relates to the number of noisy events, in which case the lower number of flights and volume of passengers transiting in Congonhas airport (21 million in 2019) compared to other studied airports, such as the Heathrow airport (75 million in 2018) in Hansell et al. [10], may partly explain these differences. It could also be due to the limited levels of aircraft noise around Congonhas airport during the night, because of local restrictions on night time flights. Results from recent large case-crossover study of CVD around Zürich Airport suggested that night-time aircraft noise may trigger acute cardiovascular mortality [44]. The risk of stroke is closely linked with age with incidence doubling with each decade after the age of 45 years. Over 70% of all strokes occur above the age of 65 [46]. As the population of Brazil (median age in 2018: 32.6 years) is much younger than the European population (median age in 2018 in the UK: 40.5), the smaller number of stroke deaths could possibly explain the lack of association observed in our study. Differences in the statistical approach and exposure assessment used, setting-specific conditions such as individual exposure determinants (e.g. housing, occupational exposure, individual risk factors), and residual confounding may also add to the variability observed in the risk estimates found in the literature.

The positive association between noise levels and CVD and CHD mortality has to be considered in the context of biological plausibility. Noise exposure has been repeatedly shown to increase blood pressure and elevate the risk of hypertension, a major risk factor for heart disease. According to the WHO review from 2019, there exists a 1.05 (95%CI: 0.95–1.17) relative risk of hypertension per 10 dB of aircraft noise [6]. Mechanisms involved in such physiological changes include both direct and indirect physiological changes involving the activation of the neuroendocrine system and increased production of stress hormones [47].

Despite growing evidence of the negative health effects of noise exposure, some methodological questions remain, in particular in relation to disentangling exposure levels and health impacts of source-specific noise levels [48]. Road traffic noise is a major source of noise and air pollution in São Paulo, with noise often exceeding the standard regulations [38]. When evaluating the effects of aircraft noise on health, extreme background noise levels could prevail over aircraft noise, raising concerns of confounding. To evaluate the contribution of each source in the total noise levels surrounding the Congonhas Airport, Scatolini and Pinto Alves (2015) measured noise levels uninterruptedly for 168 h between May and October 2009 in 15 sites around the airport [16]. They showed, that despite the high levels of background noise, aircraft noise was still an important contributor to the total noise. Héritier et al. investigated the effects of three major transportation noise sources—i.e. road, railway and aircraft—with cardiovascular mortality among 4.41 million participants of the Swiss National Cohort [43]. They observed a consistent association of all noise sources with myocardial infractions, particularly high for aircraft noise, demonstrating the independent impact of source-specific noise exposure. Another common co-pollutant is air pollution. In a follow up study, Héritier and colleagues [45] investigated the confounding effect of NO_2_ and PM_2.5_ on the same source-specific noise exposures, and vice versa. They found that the health effects of the air pollutants decreased upon adjustment for noise pollution. Again, this supports the independent effect of source-specific noise exposure and highlights the importance of correct confounder adjustment in epidemiological analyses. The inclusion of road traffic density in our analysis aimed to account for the potential confounding between different sources of noise and air pollution present in our study area. Our sensitivity analyses excluding road traffic density, suggest that road traffic related pollution is an important contributor to the mortality effects observed. However, it is important to bear in mind that traffic density is an unspecific indicator and also shows a slight spatial correlation with aircraft noise, which could lead to bias. Moreover, this approach only considers the effect of traffic noise and air pollution as a confounder and does consider the possibility of it acting synergistically or as an effect modifier as it has been suggested elsewhere [49, 50].

Our results were particularly affected by adjustment for Black and Mixed ethnicity and area-level deprivation. Evidence supporting the higher risk of CVD mortality among Black and Mixed ethnicity is unclear. The high spatial correlation with deprivation may partially explain the high contribution of this variable in our models, yet it deserves further investigation. Conversely, deprivation is a major health determinant and often positively correlated with mortality and exposure levels. In area-level epidemiological studies, it is common practise to use composite index, often developed for non-research purposes and so, its adequacy should be considered critically. In our case, the MHDI included as a component life expectancy which is directly associated to our outcomes. Nonetheless, our sensitivity analysis excluding life expectancy from the MHDI score showed almost identical results.

One remarkable finding of this study is that 60% of the study population living in the vicinities of the Congonhas Airport in São Paulo, Brazil, approximately 1.5million, was exposed to aircraft noise levels > 50 dB which is well above the WHO guideline recommendation of 45dBL_dn_ [5]. Of these, 4% lived in areas exposed to > 65 dB, classified by the Brazilian Civil Aviation Regulation n° 161 as areas that should have restricted residential and land use due to serious health concerns [18]. This puts into evidence the failure to control noise levels around airports and to set living and land use standards within the recommended noise guidelines, stressing the urgency for action. Communities’ concerns over this issue are visible and require urgent addressing. Concurrent to this study, in May 2019, São Paulo regulated the elaboration of its first road traffic noise map, the Urban Noise Map [51, 52], based on a large civil society movement, with public sectors, academic and technical bodies support. The results of the pilot study, evaluating a busy central area in the city, are now available online (http://www.mapaderuidosp.org.br/). It is aimed to act as a decision support tool for urban planning and ordering to support noise management in the city and help identify priority areas for noise reduction and preservation of areas with appropriate sound levels. Our findings highlight the importance of including aircraft noise in such evaluation tools. Failure to do so, could result in an underestimation of the areas and population affected by exceeding noise levels.

Finally, we recognize several limitations of our approach. Firstly, the exposure assessment was based on limited noise measurements and an acoustic model for 2017, combining both daytime and night-time noise. Personal exposure to noise levels is different at night-time and so, our model fails to distinguish such differences. Moreover, disruptions of the night cycle, either actively (i.e. conscious disruption of sleep) or passively, have been shown to have a larger impact on health, by causing a more pronounced increase in levels of stress hormones and vascular oxidative stress [53, 54]. However, only few flights take place overnight at Congonhas airport (approx. 8%) since flight restrictions are in place between 11 pm and 6 am. And so, we would expect its effects to be small. Secondly, the use of 5 dB categories is not standard which may hinder comparability with other studies and affect the power to detect associations. To facilitate international comparison, we estimated risk associated to 10 dB increases in noise exposure, which showed similar trends (yet not significant). Thirdly, other sources of noise and presence of co-pollutants such as air pollution may also affect our results. We incorporated data on road traffic density to address the confounding effect of noise and air pollution from road traffic. Finally, we did not account for other confounders including housing characteristics (e.g. construction type, window glazing, presence of air conditioning) or individual level covariates, including lifestyle factors such as smoking, and morbidity, as they were not available. Although we believe that the use of lung cancer as a proxy for smoking is appropriate in the absence of tobacco data – supported for example by the strong correlation identified between spatial smoking patterns from survey data with lung mortality smoothed estimates in a Swiss study [55] - the strength of this association in a large city such as São Paulo should be further investigated.

Overall, we believe our study adds to the existing knowledge in the field of noise exposure epidemiology and adds a new perspective on a LMIC setting. It also is the first study of the health impact of aircraft noise exposure in Brazil. It covers a large study population of 3.5 million residents with good exposure contrast and high-quality death records. We have been able to adjust for major covariates such as socioeconomic development, ethnic composition, smoking and road traffic density, albeit at small-area level rather than at the individual level. By using small geographies, such as census tracts, the small-area design attempts to provide a closer estimation to individual-level risks and to minimise the ecological fallacy [56]. Although the presence of residual confounding and ecological bias need to be considered further, small-area studies therefore offer a valuable epidemiological approach to assess health risks posed by environmental pollutants such as aircraft noise, as previously demonstrated in a study of the health impact of aircraft noise near Heathrow airport, London, UK [10].

## Conclusions

In conclusion, our small-area study, conducted in a South American megacity, provided suggestive evidence that exposure to aircraft noise is associated with increased risk of CVD and CHD deaths. Aircraft noise pollution around the Congonhas airport is much higher in areas near the airport than WHO recommendations and public health measures to reduce exposure would nevertheless help reducing potential health impacts in local populations. In light of our findings, we recommend future noise models to include multiple noise sources to create comprehensive population noise exposure profiles of the city. As an example, we suggest to expand the municipality-wide Urban Noise Map currently in development stages to include aircraft noise. Such a tool would prove extremely useful for research purposes, for the evaluation of urban interventions, and for the formulation of public policies necessary to guarantee people health that live in areas exposed to high-level decibels.

## Supplementary Information

**Additional file 1: Fig. S1.** Noise contours***.*** A. L_dn_ noise contours > 65 dB as provided by the Brazilian Airport Infrastructure Company (INFRAERO) for Congonhas airport, São Paulo, Brazil. B. Noise contours (yellow) as estimated by Prof. Jules G. Slama (personal communication). Districts selected for being partially or entirely exposed to noise levels are shown in dark blue. The grey outline illustrates the boundaries of the Municipality of São Paulo. **Fig. S2.** Maps of covariates within our study area, São Paulo, Brazil. Spatial distribution of: A. the quintiles of the Index of Human Development (IHD); B. Proportion of Black and Mixed population; C. proportion of East Asian population; D. Quintiles of total traffic density as a proxy for air pollution E. Smoothed lung mortality risk; and F. Posterior probability of lung mortality risk. **Fig. S3.** Scatter plot between the standard MHDI 2010 score and the modified MHDI 2010**.** The modified MHDI was re-calculated excluding the life expectancy indicator using a geometric mean. The red line, the fitted linear regression. **Fig. S4.** Spatial distribution the standard and modified MHDI 2010. The standard (left) and modified (right) MHDI for both the continuous score (top) and quintiles classification (bottom). **Fig. S5-S9.** Correlations between covariates. The correlation coefficient and *p*-value of the Cramer’s V-square test are shown. V Cramer = 1 denotes strong association and V Cramer = 0 denotes weak association. Bar plots indicate the number of census tracts belonging to each category for a given covariate against each of the other covariates.

**Additional file 2: Table S1.** Model selection*.* Information regarding the forward stepwise regression models fitted including model name, description and Akaike information criterion (AIC) for cardiovascular diseases (CVD), stroke and coronary disease. Shaded those presented in the manuscript. Bolded the model with the best fit; shaded, models fully presented in the full text.

**Additional file 3: Table S2.** Relative risk (RR) and 95% confidence intervals (CI) for the association between CVD, stroke and CHD with 10 dB increase in noise. Adjusted model only (i.e. Adjusted for age and sex (standardization) **+** smoking proxy + IDHM 2010 + Black and Mixed ethnicity + road traffic density). Trends estimated using WHO standard methods, i.e. generalized least square estimation.

## Data Availability

Outcome data. The health data that support the findings of this study are not publicly available due the need of protection of individual information. Aggregated data by larger areas are however available from the Epidemiology and Information Department of the Municipal Health Secretariat of São Paulo (CEInfo/SMS-SP), https://www.prefeitura.sp.gov.br/cidade/secretarias/saude/epidemiologia_e_informacao/ [20]. Noise data. The noise exposure data that support the findings of this study are available from the Brazilian airport Infrastructure Company and by direct communications with Prof. Slama, but restrictions may apply to the availability of these data. Data are however available from the authors upon reasonable request and with permission of the Brazilian airport Infrastructure Company and Prof. Slama. Population data. The age- and sex-specific population data for census tracts in our study area and modelled by the co-authors, are available from the corresponding author on reasonable request. The district-level age- and sex specific population projections and the total census tract population counts used to estimate this, are available from the State-wise System for Data Analysis (SEADE) Foundation *Projecoes Populacionais,* http://produtos.seade.gov.br/produtos/projpop [18], and from the 2010 Census Tract Population repository provided by the Brazilian Institute of Geography and Statistics (IBGE),https://www.ibge.gov.br/en/statistics/social/population/22836-2020-census-censo4.html?=&t=o-que-e [19], respectively. Ethnic composition. The dataset analyzed during the current study is available in the 2010 Brazilian Population Census repository, https://www.ibge.gov.br/en/statistics/social/population/22836-2020-census-censo4.html?=&t=o-que-e [19]. Smoking proxy and traffic density. The datasets used and/or analyzed during the current study are available from the corresponding author on reasonable request.

## Abbreviations

AIC: Akaike information criterion
ANAC: Brazilian national civil aviation agency
BCR: Biomedical research centre
BYM: Besag-York-Molliè
CEInfo/SMS-SP: Epidemiology and information department of the municipal health secretariat of São Paulo
CET: *Companhia de Engenharia de Tráfego*
CHD: Coronary heart diseases
CI: Confidence interval
CVD: Cardiovascular diseases
dB: Decibels
DEM: *Departamento de Engenharia Mecânica *
FAA: Federal aviation administration
FAPESP: *Fundação de Amparo à Pesquisa do Estado de São Paulo*
HDI: Human development index
HYENA: HYpertension and exposure to noise near airports
IBGE: Brazilian institute of geography and statistics
iCAR: Intrinsic conditional autoregressive
ICD-10: International classification of diseases 10th edition
INFRAERO: Brazilian airport infrastructure company
IQR: Interquartile range
Ldn: Day-night average noise level
LMIC: Low and middle-income countries
MHDI: Municipal human development index
MRC: Medical research council
NIHR: National institute for health research
RR: Relative risk
SAHSU: Small area health statistics unit
SEADE: State-wide system for data analysis
SPRINT: São Paulo researchers in international collaboration
UNDP: United Nations development program
WHO: World health Organization
