# Supplementary material for: Risk of cardiovascular mortality, stroke and coronary heart mortality associated with aircraft noise around Congonhas airport, São Paulo, Brazil: a small-area study
Source: Environ Health. 2021 May 13;20:59. doi: 10.1186/s12940-021-00746-7 (PMC8120910; doi:10.1186/s12940-021-00746-7)
Supplement: Supplementary file 1 — Additional file 1: Fig. S1. Noise contours. A. Ldn noise contours > 65 dB as provided by the Brazilian Airport Infrastructure Company (INFRAERO) for Congonhas airport, São Paulo, Brazil. B. Noise contours (yellow) as estimated by Prof. Jules G. Slama (personal communication). Districts selected for being partially or entirely exposed to noise levels are shown in dark blue. The grey outline illustrates the boundaries of the Municipality of São Paulo. Fig. S2. Maps of covariates within our study area, São Paulo, Brazil. Spatial distribution of: A. the quintiles of the Index of Human Development (IHD); B. Proportion of Black and Mixed population; C. proportion of East Asian population; D. Quintiles of total traffic density as a proxy for air pollution E. Smoothed lung mortality risk; and F. Posterior probability of lung mortality risk. Fig. S3. Scatter plot between the standard MHDI 2010 score and the modified MHDI 2010. The modified MHDI was re-calculated excluding the life expectancy indicator using a geometric mean. The red line, the fitted linear regression. Fig. S4. Spatial distribution the standard and modified MHDI 2010. The standard (left) and modified (right) MHDI for both the continuous score (top) and quintiles classification (bottom). Fig. S5-S9. Correlations between covariates. The correlation coefficient and p-value of the Cramer’s V-square test are shown. V Cramer = 1 denotes strong association and V Cramer = 0 denotes weak association. Bar plots indicate the number of census tracts belonging to each category for a given covariate against each of the other covariates. [file 12940_2021_746_MOESM1_ESM.docx]

**Additional File** **1. Supplementary Figures**


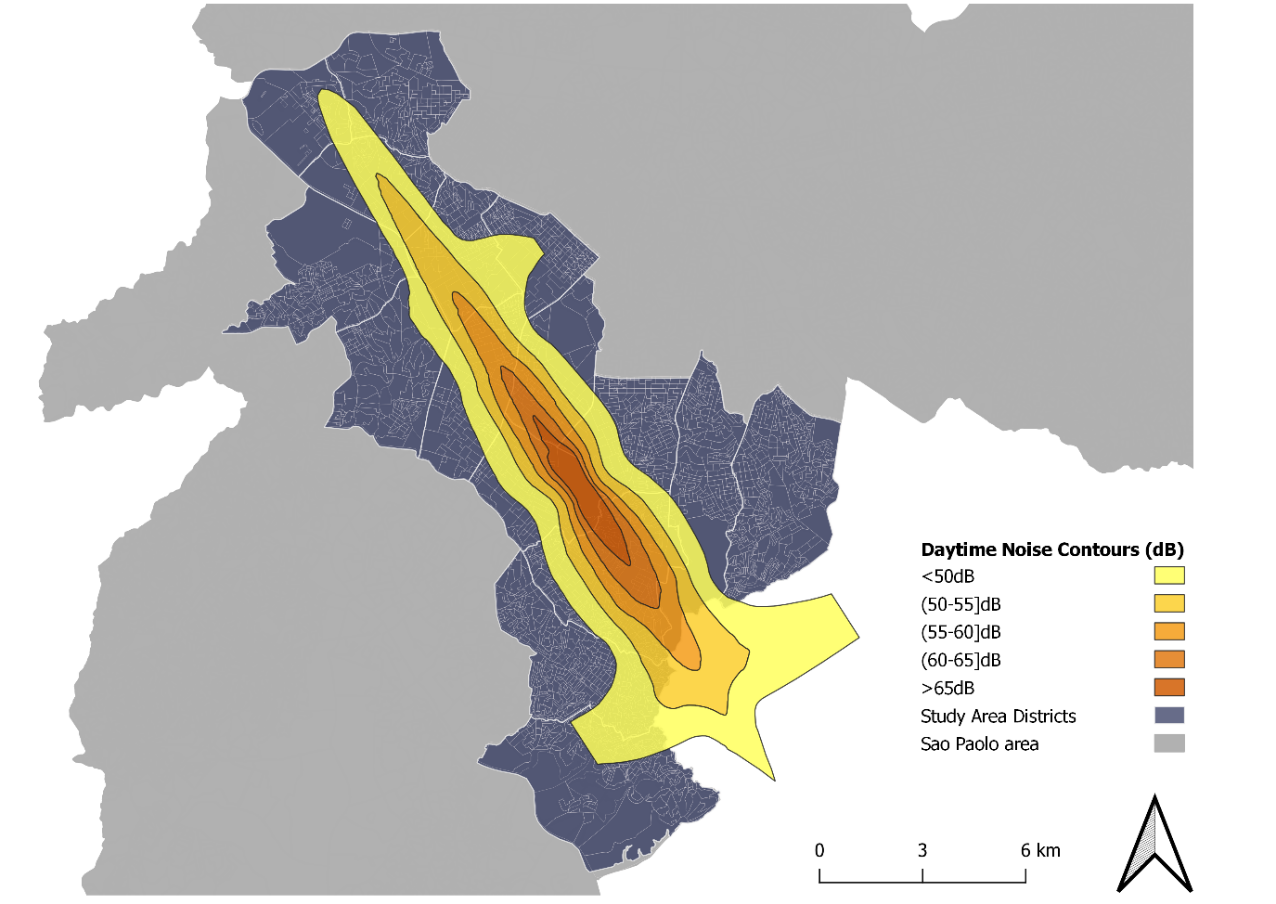

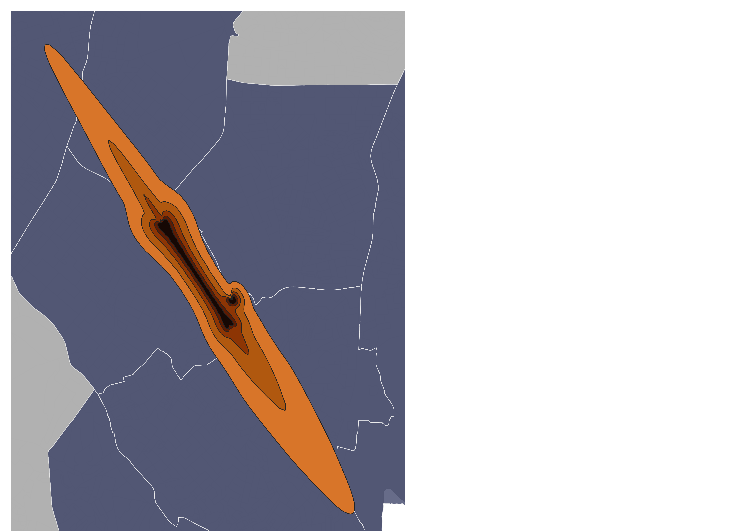


**A**

**B**


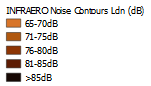


Figure S1: Noise contours.

| 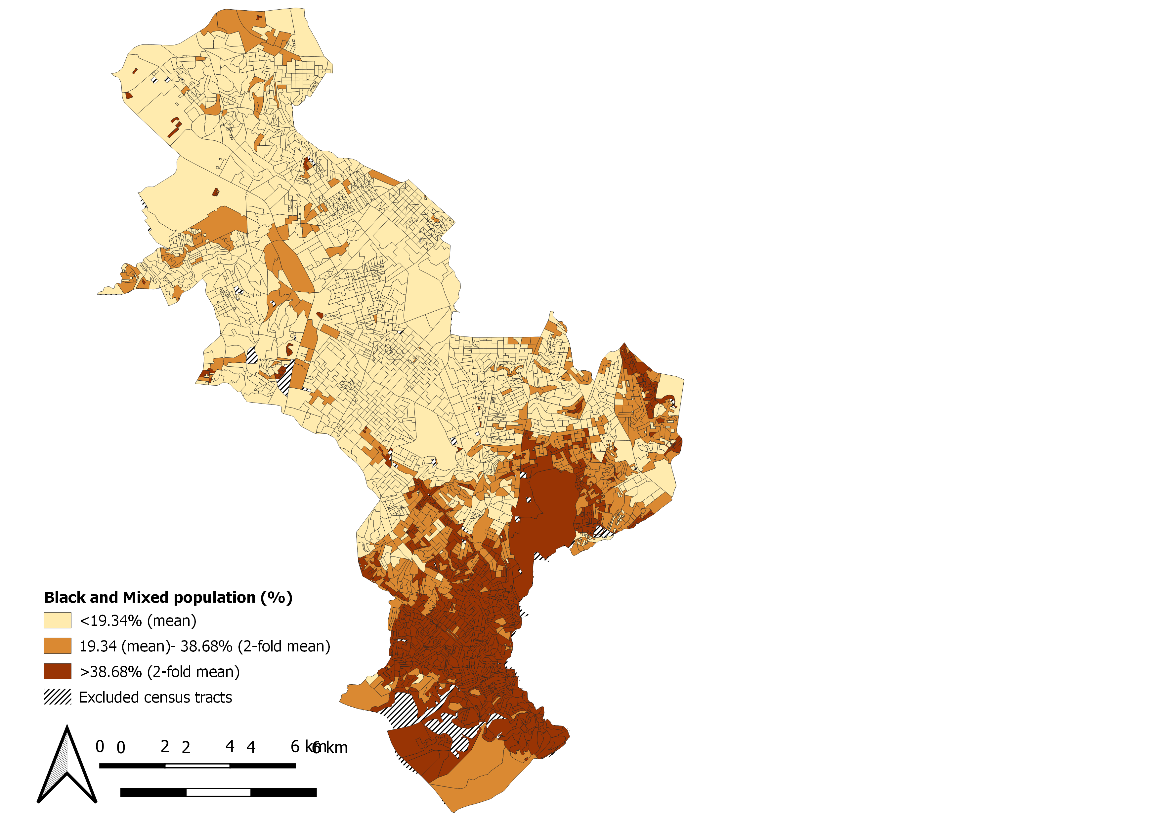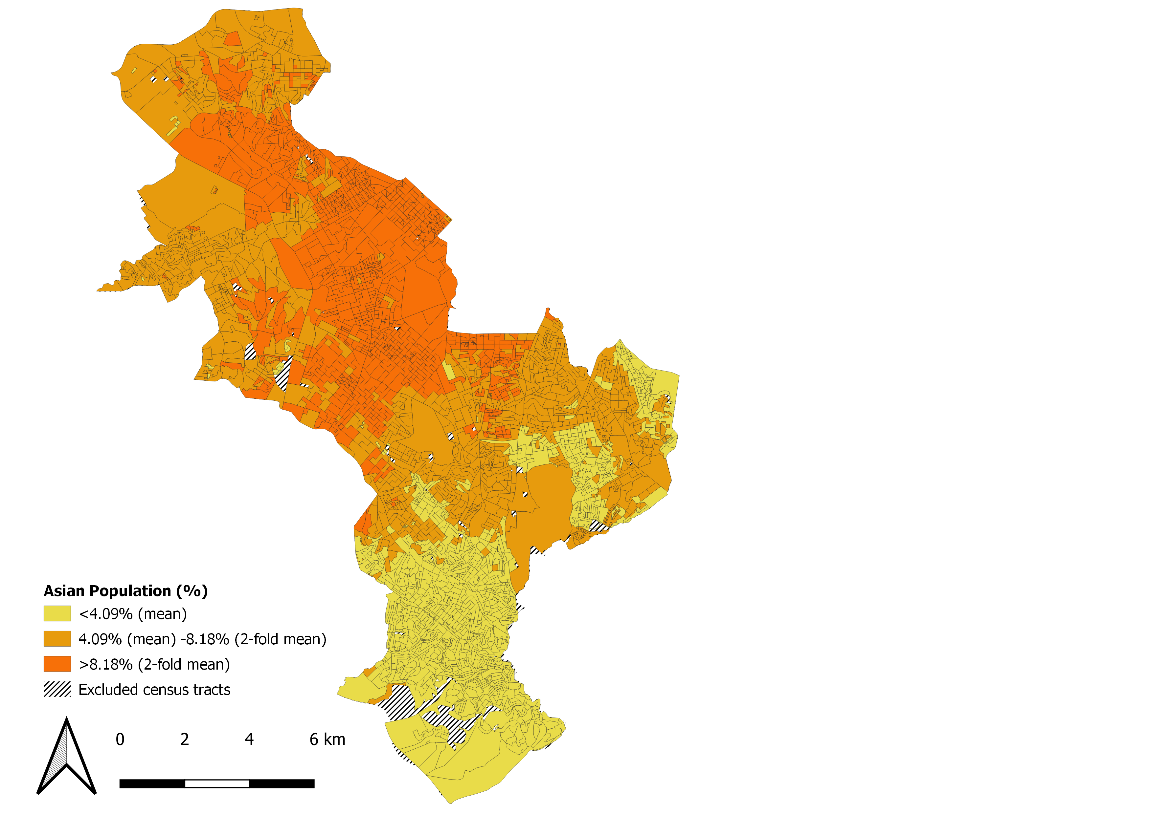 **A**  **B**  **C** 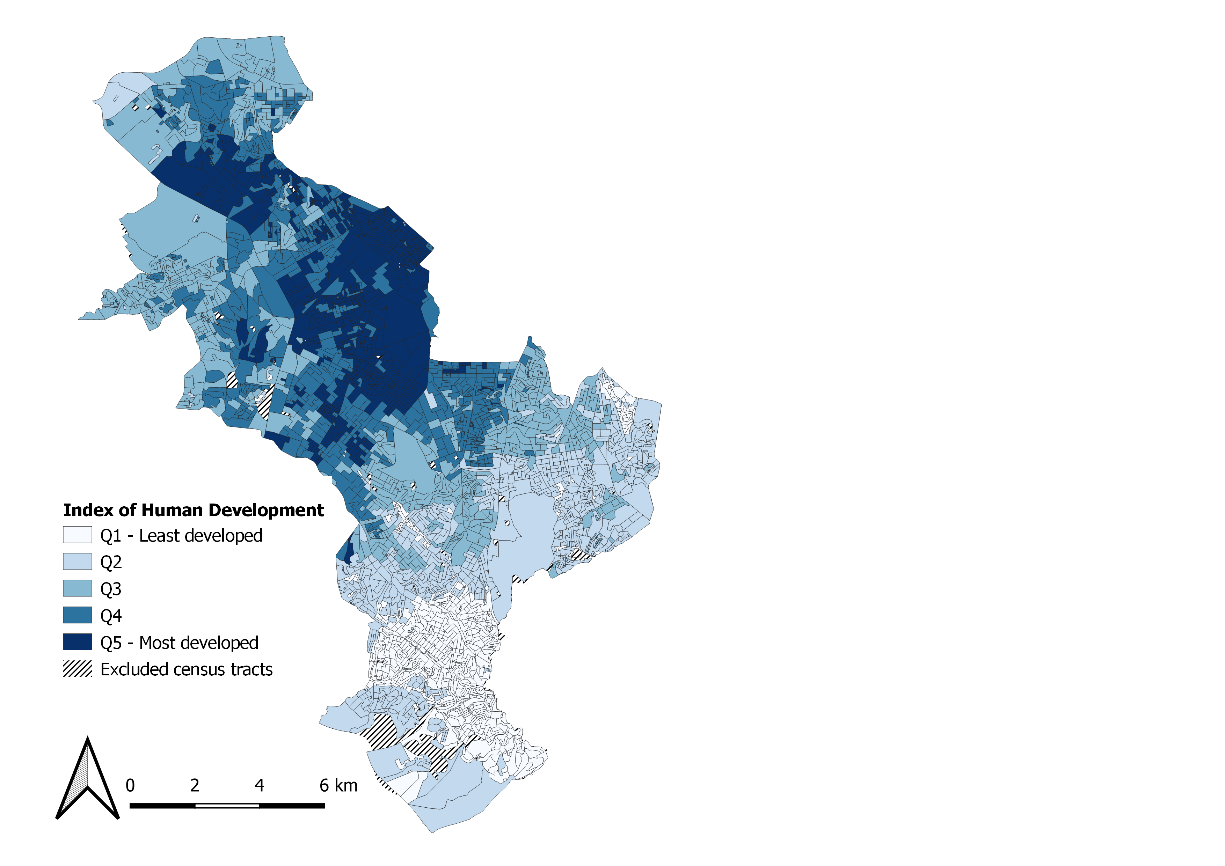 Figure S2. Maps of covariates within our study area, São Paulo, Brazil. | | |
| --- | --- | --- |
| **D**  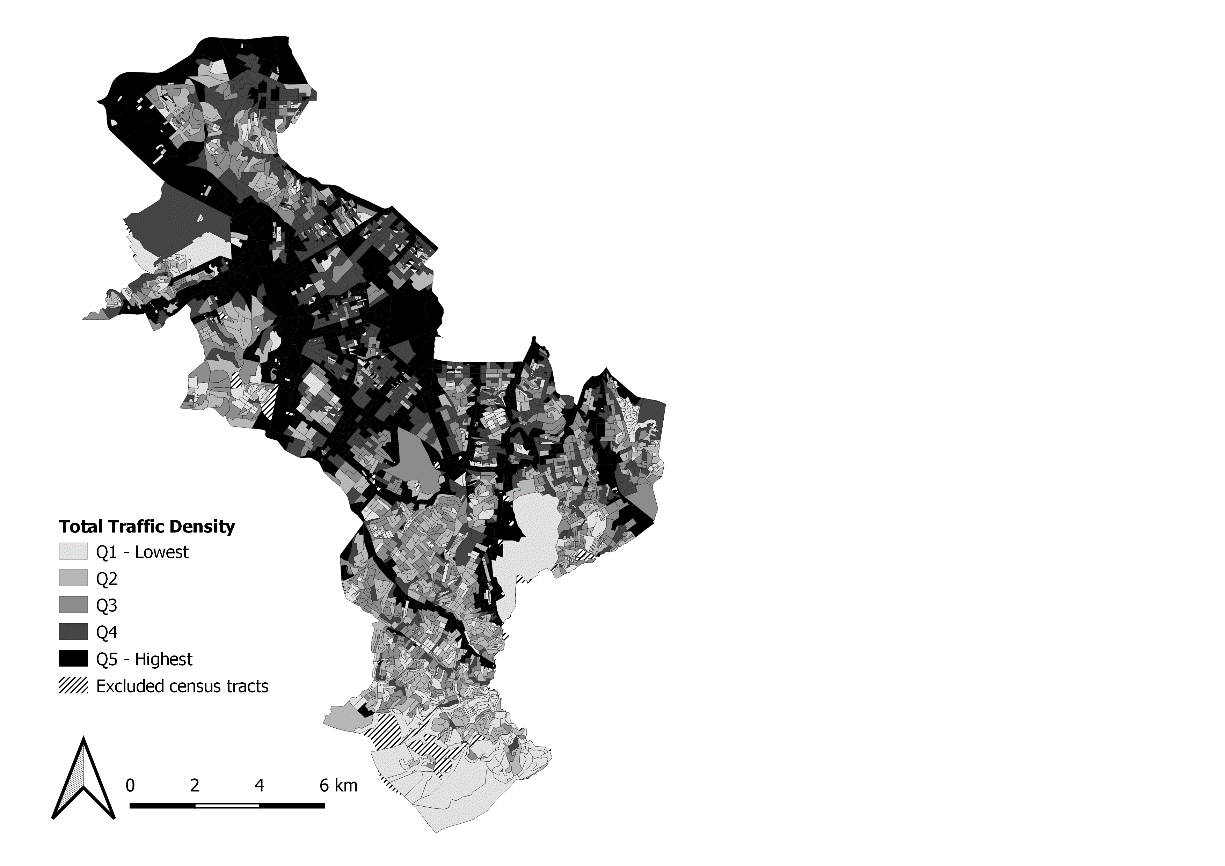 | **E**  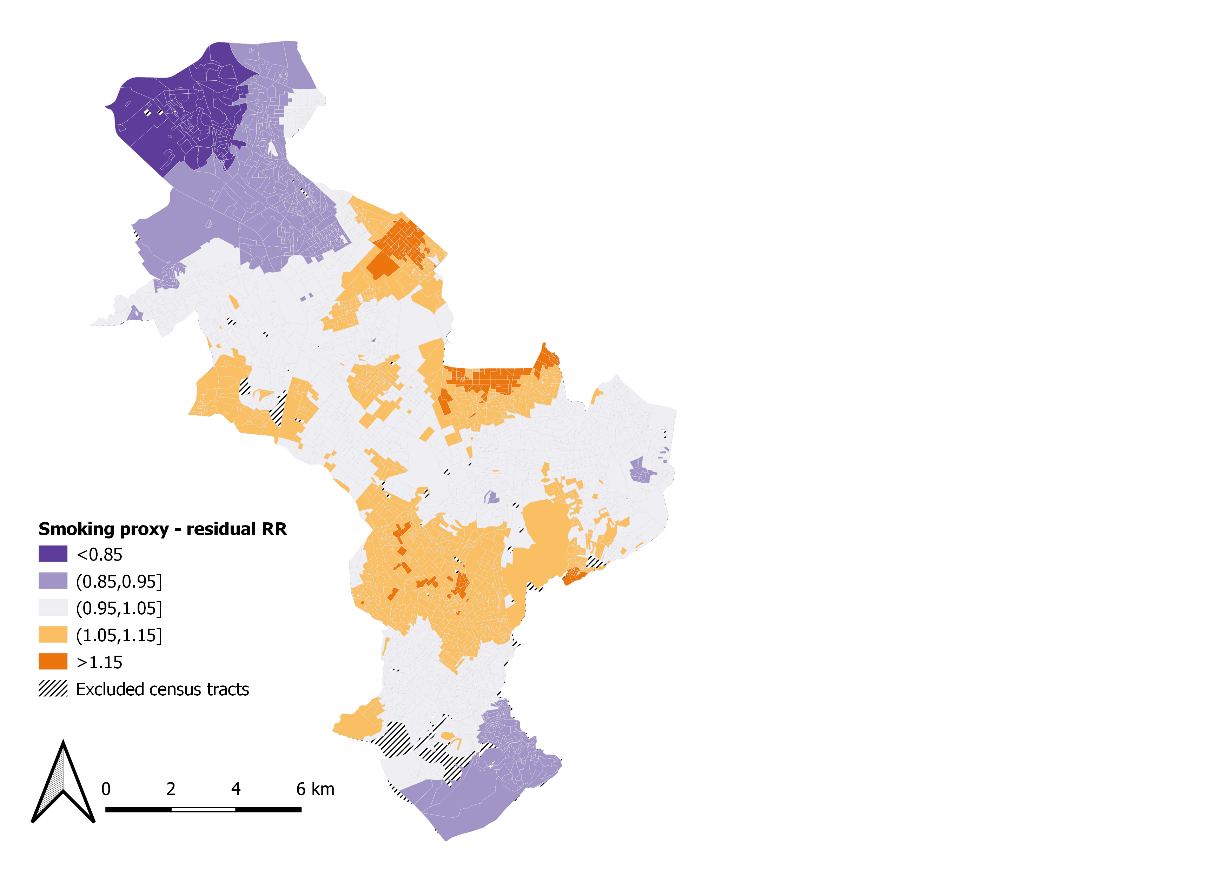 | **F**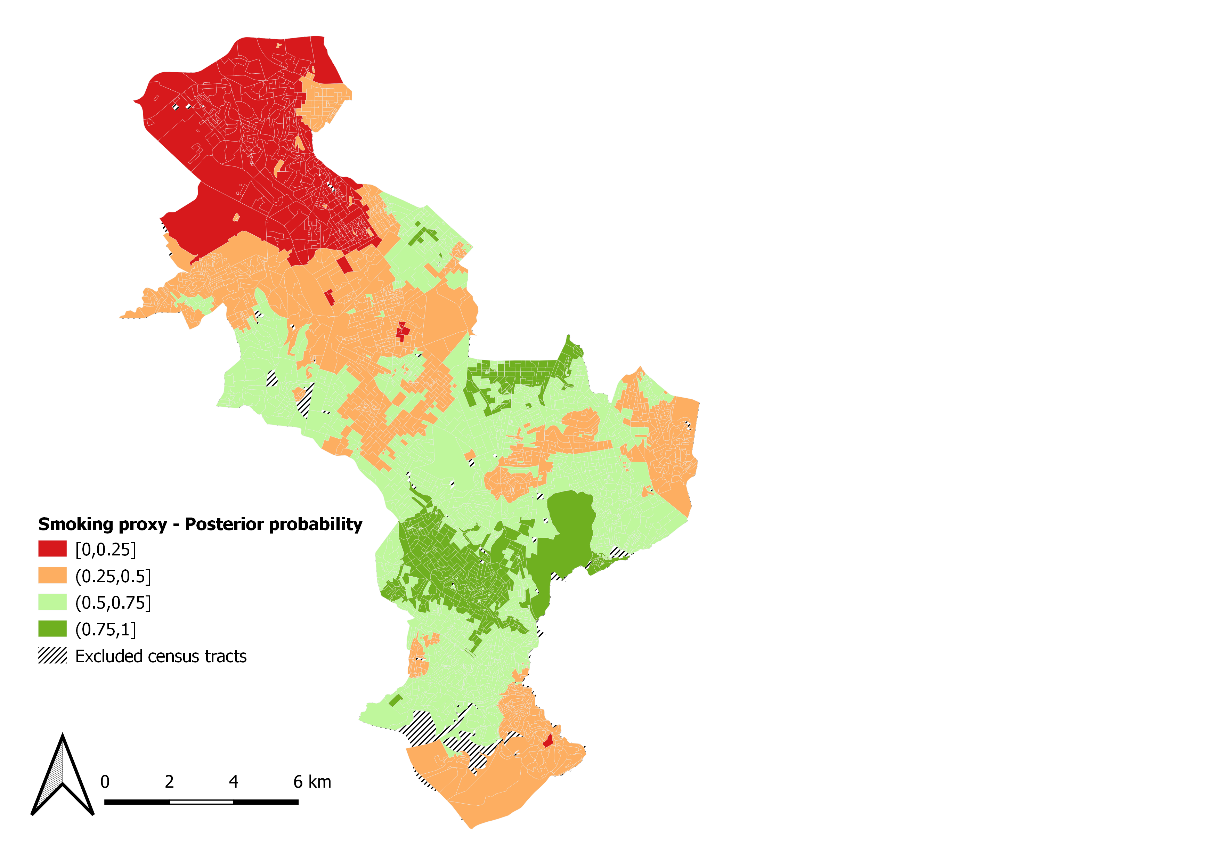 |

Figure S2 (cont.): Maps of covariates within our study area, São Paulo, Brazil.

.

**Modified MHDI 2010 (excl. life expectancy)**

**Standard MHDI 2010**


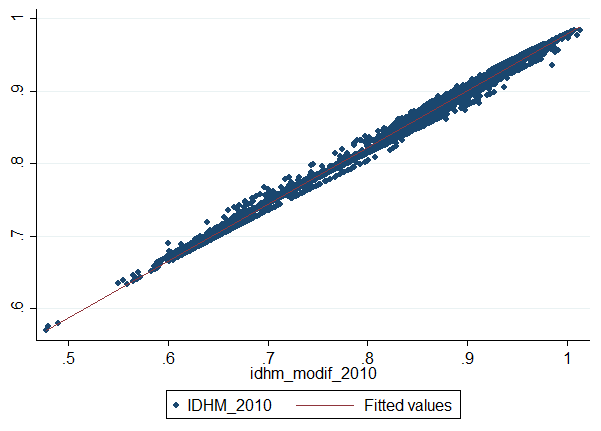


Figure S3. Scatter plot between the standard MHDI 2010 score and the modified MHDI 2010,

Figure S4. Spatial distribution the standard and modified MHDI 2010.


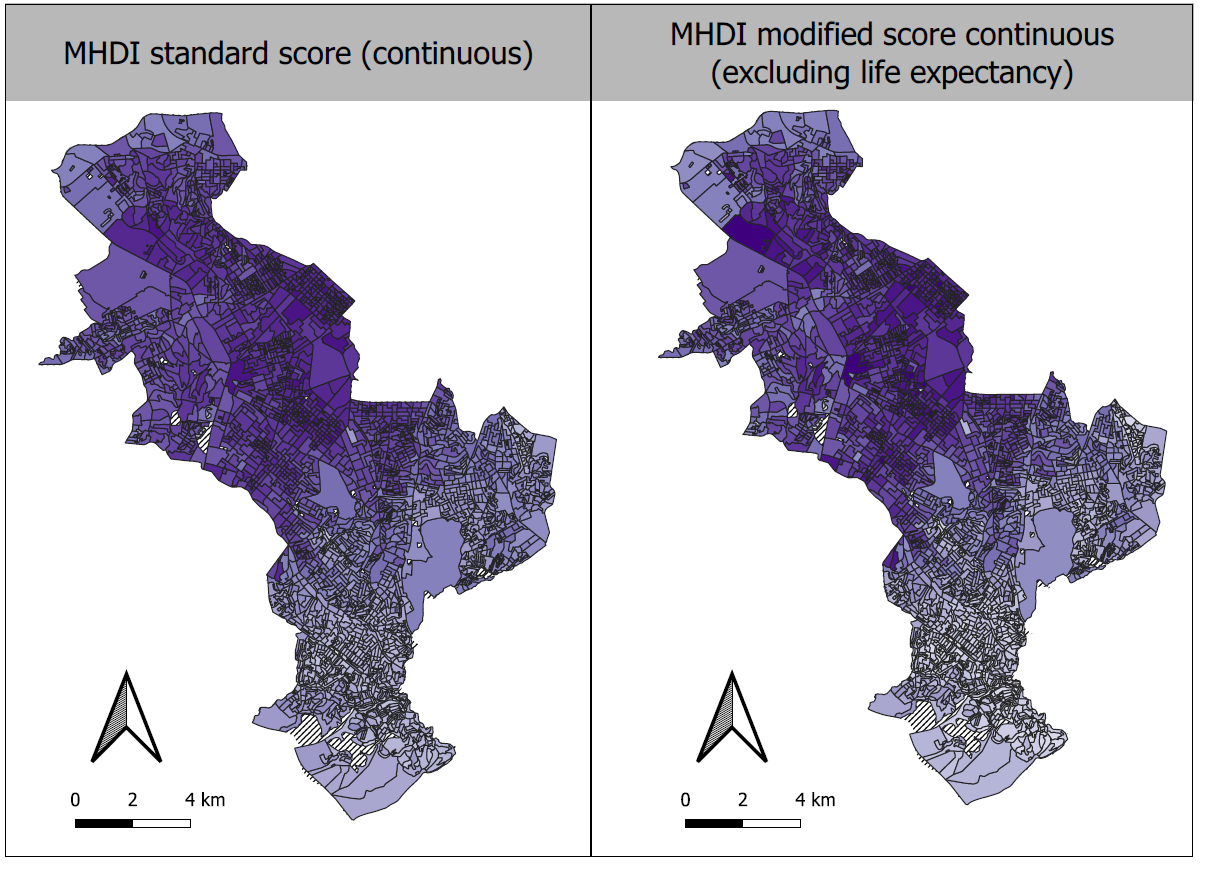

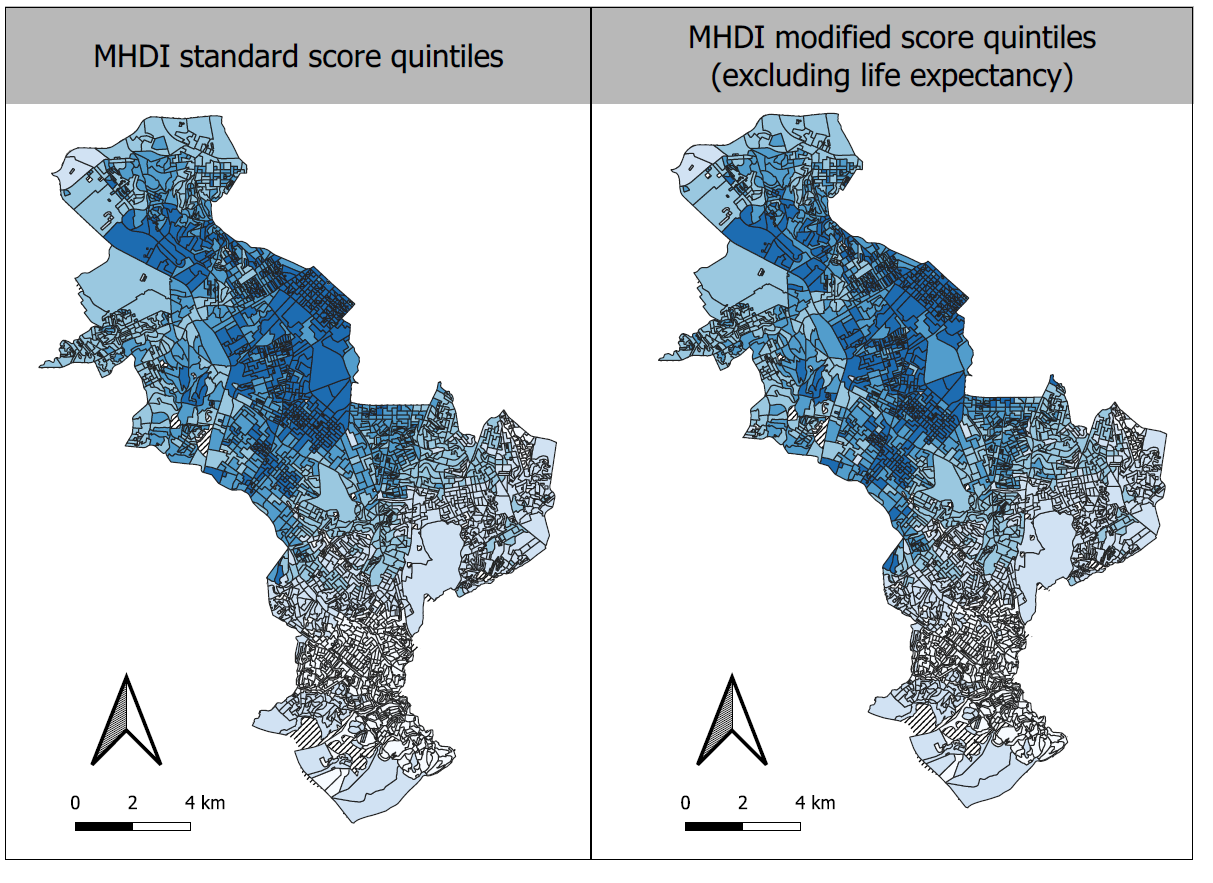

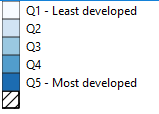

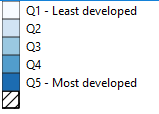

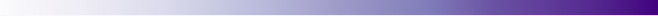

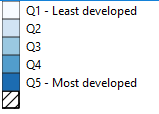

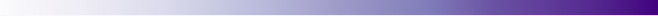

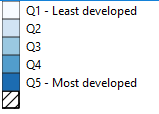


**Excluded census tracts**

**Excluded census tracts**


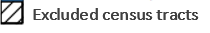

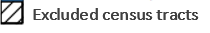

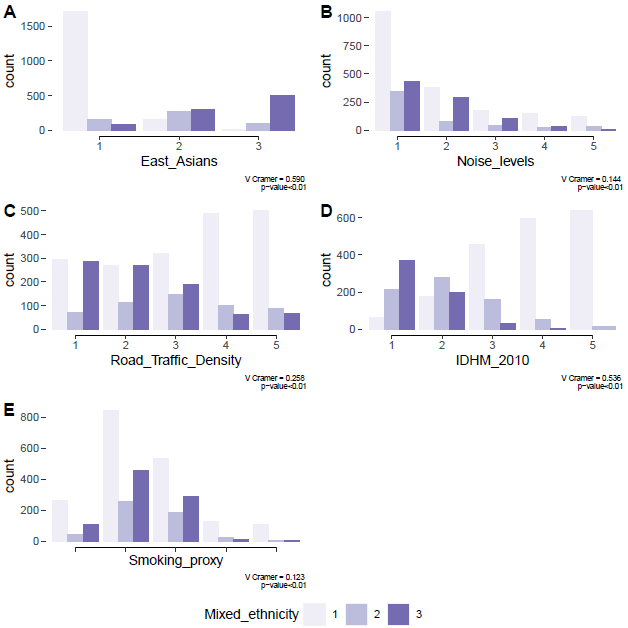


MHDI 2010

Figure S5: Correlations between mixed ethnicity and other covariates.


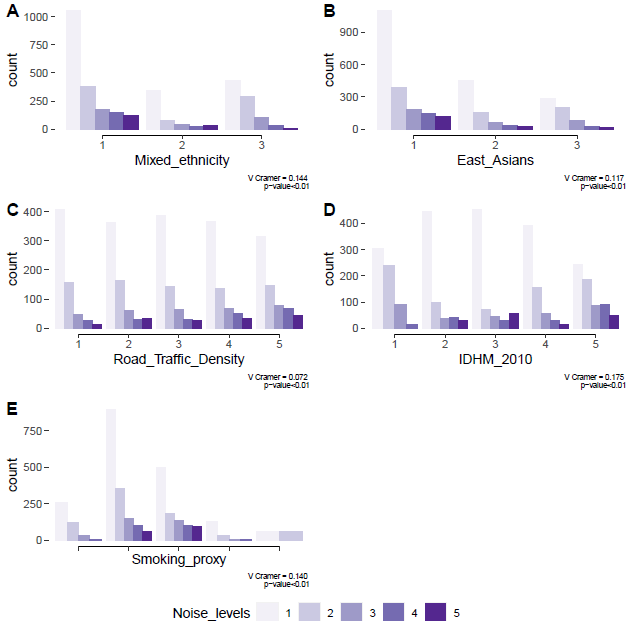


MHDI 2010

Figure S6: Correlations between noise and covariates.


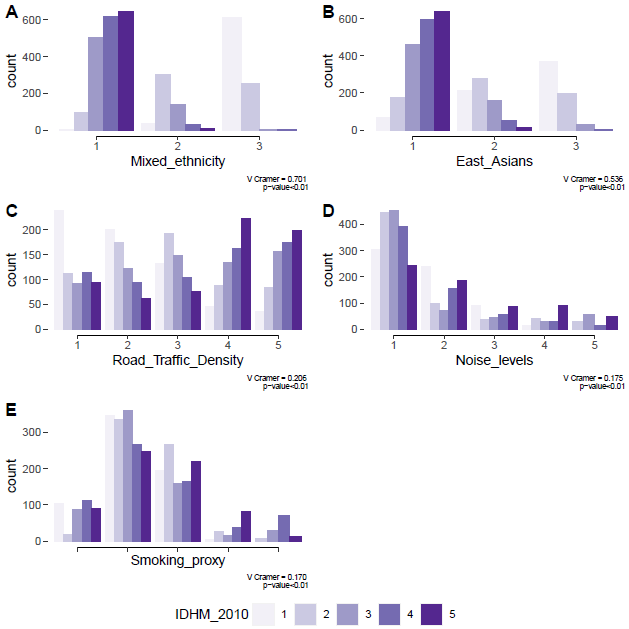


MHDI 2010

Figure S7: Correlations between socio-economic status and other covariates.


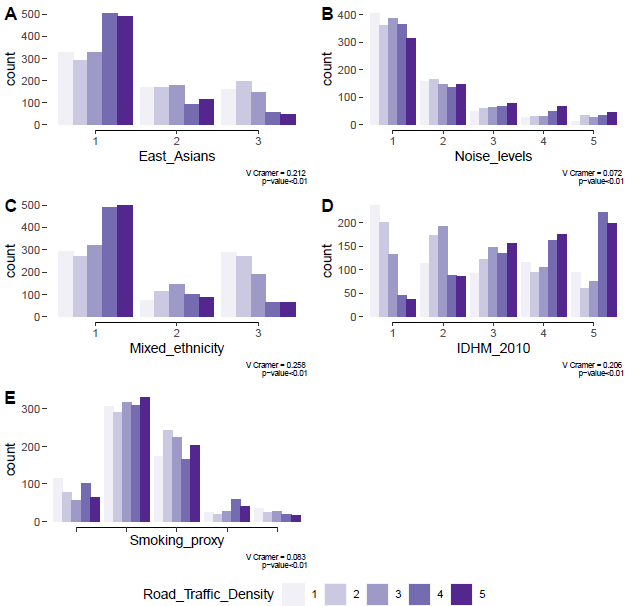


MHDI 2010

Figure S8: Correlations between road traffic density and other covariates.


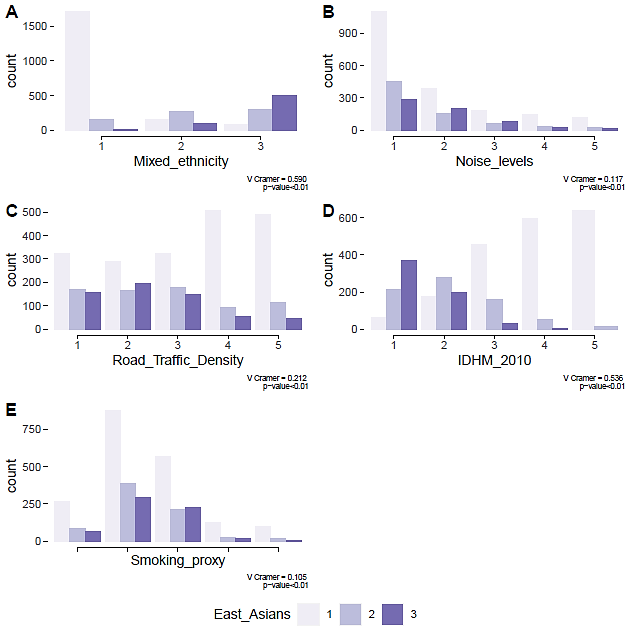


MHDI 2010

Figure S9: Correlations between East Asian ethnicity and other covariates.
